# Differential Diagnosis

**Published:** 1911-04

**Authors:** 


**Differential Diagnosis.** Presented through an Analysis of 383 cases. By **Richard C. Cabot,** M.D., Assistant Professor of Clinical Medicine, Harvard Medical School. Octavo of 753 pages. Illustrated. Philadelphia and London: W. B. Saunders Company. 1911. (Cloth, $5.50, net.)

The book treats in separate chapters of pain, general and local, fevers, chills, coma, convulsions, weakness, cough, vomiting, hematuria, dyspnea, jaundice and nervousness. It is quite an unusual monograph. We have many works on medical diagnosis, in which the matter of differential diagnosis is a prominent topic. Cabot's aim is different, his purpose is to make plain the diagnostic value of certain of the more frequent signs or symptoms of disease, or the complaints of those who think they are ill.

We cannot congratulate the author upon his selection of a phrase to indicate the precise character of his book "the presenting symptom." We freely admit that it is quite imperative that the obstetrician should be able to diagnose the presenting part (symptom) at least to tell whether the vertex or the breach is presenting, but to give this delicate situation the prominence of a virtual subtitle of a work on medical diagnosis is to forget the dignity of the occasion.

The author devotes extended space to a consideration of the evidential value of pain, general and local.—12 of 23 chapters, 388 of 733 pages, 212 of 383 illustrative cases. The 212 cases devoted to this major part of our book, include 94 different diagnoses. The order of the frequency of the different diseases of this group is highly suggestive. We find of tuberculosis 20 cases, syphilis 15, cancer 12, gall-stones 8, renal stones 7, neuroses 7, typhoid fever 6, ovarian cysts 5, pericarditis 4, pneumonia 4, constipation 4, alcoholism 4. The remaining 116 cases are divided between 85 different diagnoses

This would seem to show that pain, general or local, has not much evidential value in the way of the differential diagnosis of disease.

We observe with regret, that in the study of his cases, the author seems to make little of errors in eating and drinking as factors in etiology. Also that his careless habit of testing the urine makes him commit the blunder of crediting the kidneys with normal or negative urine only a few days prior to the death of the patient from chronic nephritis. To print such records speaks volumes for the courage and honesty of the author. The book is well worth studying. H. R. H.

